# Self-Medication in Mexican Pediatric Patients

**Published:** 2019-12

**Authors:** Mario. I. ORTIZ, Beatriz MARTÍNEZ JIMÉNEZ, Ricardo RAMÍREZHERNÁNDEZ, María de Los Ángeles CASTELÁN-CAMPOS, Raquel CARIÑOCORTÉS, Héctor A. PONCE-MONTER

**Affiliations:** 1. Department of Medicine, Center for Research on Reproductive Biology, Autonomous University of Hidalgo State, Pachuca, Hidalgo, Mexico; 2. General Hospital of the Secretary of Health Hidalgo, Pachuca, Hidalgo, Mexico; 3. Dr. Daniel Mendez Hernández Hospital of Infectology, La Raza Medical Center, Mexico City, Mexico; 4. Dr. Juan N. Navarro Children's Psychiatric Hospital, Mexico City, Mexico

## Dear Editor-in-Chief

Self-medication (SM) is the use of non-prescribed medicines by individuals to treat self-recognized disorders or symptoms (1, 2). SM can include the use of conventional medicines (CM) or a wide range of complementary and alternative medicine (CAM). There are advantages and disadvantages of SM (1–3). The prevalence of SM in children ranges between 7.1 to 58.8% (3–7). In general, parents or legal guardians perform the SM (3–7).

We aimed to evaluate the characteristic and frequency of SM (CM and CAM) of Mexican pediatric patients in a hospital outpatient department. The Ethics and Investigation Committees approved the study protocol, and the study was performed according to the guidelines delineated by the Declaration of Helsinki.

A total of 941 parents or legal guardians with a mean age of 31.7 (±8.7) yr were evaluated. The mean age of the children was of 56.6 (±81.6) months. Five hundred forty children were male (57.4%). Respiratory (24.9%), nervous and musculoskeletal system (18.1%), gastrointestinal (13.6%), skin (8.8%), and psychological or psychiatric (7.8%) diseases were the main consultation diagnoses. Three-hundred-fifty-seven (37.9%) parents or legal guardians gave CM to their children before going to the hospital. Five-hundred-twenty-two CMs were administered to the children, with an average of 1.46 medicines/child, being the antipyretics/non-steroidal anti-inflammatory drugs (NSAIDs) the main group. The commonest source about the information of the medicines was by old prescription in 237 (66.4%) cases and in 88 (24.6%) cases the drugs were indicated by parents or legal guardians. Table 1 shows the CM groups used by the parents or guardians of the children.

**Table 1:** Number of medications (CM) by pharmaceutical group.

***Variable***	***n***	***%***
Antipyretic and non-steroidal anti-inflammatory drugs	389	74.5
Paracetamol	289	74.3
Metamizol	34	8.7
Nimesulide	28	7.2
Others	38	9.8
Ear, nose, throat, respiratory medications	65	12.5
Ambroxol	36	55.4
Amantadine, chlorpheniramine, paracetamol	7	10.8
Loratadine	6	9.2
Others	16	24.6
Antimicrobial medicines	50	9.4
Trimethoprim-sulfamethoxazole combination	15	30.0
Ampicillin	12	24.0
Amoxicillin	12	24.0
Others	11	22.0
Gastrointestinal medicines	16	3.1
Butylscopolamine	7	43.8
Bismuth subsalicylate	2	12.5
Others	7	43.8
Others	2	0.4

CM = Conventional medicines

Three-hundred-one (32.0%) parents or legal guardians gave CAM to their children before going to the hospital. Five-hundred-two CAMs were administered to the children, with an average of 1.67 CAM/child. Table 2 shows the CAMs used by the parents or legal guardians of the children, being the chamomile infusion the main administered CAM.

**Table 2:** Number of CAMs administered in children by parents or legal guardians (n=301)

***Variable***	***n***	***%***
Chamomile (*Matricaria chamomilla*) infusion	212	42.2
Peppermint (*Mentha × piperita* L. ) infusions	93	18.5
Cinnamon (*Cinnamomum verum*) infusions	28	5.6
Honey with lemon beverage	16	3.2
Lemon grass (*Cymbopogon citratus*) infusions	15	3.0
Leaf of Guava (*Psidium guajava* L. ) infusions	9	1.8
Melissa (*Melissa officinalis*) infusions	9	1.8
Oregano (*Origanum onites* L ) infusions	8	1.6
Mullein (*Verbascum Thapsus*) infusions	8	1.6
Eucalyptus (*Eucalyptus tereticornis*) infusions	8	1.6
Honey alone	8	1.6
Bougainvillea (*Bougainvillea spectabilis*) infusions	7	1.4
Olive oil	6	1.2
Prune (*Prunus domestica*) infusions	5	1.0
Cedar tea	5	1.0
Wormwood (*Artemisia absinthium* L ) infusions	4	0.8
Rue (*Ruta graveolens*) infusions	4	0.8
Others	57	11.4

CAM = Complementary and alternative medicine.

The commonest source about the information of the CAMs was by grandparents or relatives in 197 (65.4%) cases and in 67 (22.3%) cases the CAMs were indicated by parents or legal guardians. With respect to the annual frequency of SM (last 12 months), 626 (66.5%) parents self-medicate with CM and 435 (46.2%) with CAM.

Parents or legal guardians used CMs in SM with a mean of 3.5 ± 2.9 times/year. Parents or legal guardians used CAMs with a mean of 3.9 ± 3.4 times/year. Four-hundred-twenty-four (45.1%) parents or legal guardians administered CM in self-decision to children during the last year and 367 (39.0%) parents or legal guardians managed CAMs to children. The annual means of the administration of CMs to children was of 2.9 ± 2.2 times/year and of CAMs was of 2.9 ± 2.4 times/year. Significant correlation coefficients were found for the SM (CM and CAM) frequency of parents with the frequencies of CM and CAM administered to the children (*P*<0.05).

We found data on common CM and CAM used as SM in Mexican children. Parents or legal guardians are responsible for the care of their children and they must consider the security of these interventions to reduce adverse o deleterious effects in children.
